# Visualizing the antivascular effect of bortezomib on the hypoxic tumor microenvironment

**DOI:** 10.18632/oncotarget.5300

**Published:** 2015-09-23

**Authors:** Xiaorong Sun, Ellen Ackerstaff, Fuqiu He, Ligang Xing, Hung Tsung Hsiao, Jason A. Koutcher, C. Clifton Ling, Gloria C. Li

**Affiliations:** ^1^ Department of Radiology, Shandong Key Laboratory of Radiation Oncology, Shandong Cancer Hospital and Institute, Jinan, Shandong, China; ^2^ Department of Radiation Oncology, Memorial Sloan Kettering Cancer Center, New York, NY, USA; ^3^ Department of Medical Physics, Memorial Sloan Kettering Cancer Center, New York, NY, USA; ^4^ Department of Radiation Oncology, Shandong Key Laboratory of Radiation Oncology, Shandong Cancer Hospital and Institute, Jinan, Shandong, China; ^5^ Current address: Department of Anesthesiology, E-Da Hospital, Yanchau District, Kaohsiung, Taiwan

**Keywords:** bortezomib, hypoxia, endothelial cell, dynamic contrast enhanced magnetic resonance imaging

## Abstract

Bortezomib, a novel proteasome inhibitor, has been approved for treating multiple myeloma and mantle cell lymphoma and studied pre-clinically and clinically for solid tumors. Preferential cytotoxicity of bortezomib was found toward hypoxic tumor cells and endothelial cells *in vitro*. The purpose of this study is to investigate the role of a pretreatment hypoxic tumor microenvironment on the effects of bortezomib *in vitro* and *ex vivo*, and explore the feasibility of dynamic contrast enhanced magnetic resonance imaging (DCE MRI) to noninvasively evaluate the biological effects of bortezomib. It was shown *in vitro* by Western blot, flow cytometry, and ELISA that bortezomib accumulated HIF-1α in non-functional forms and blocks its hypoxia response in human colorectal cancer cell lines. *Ex vivo* experiments were performed with fluorescent immunohistochemical staining techniques using multiple endogenous and exogenous markers to identify hypoxia (pimonidazole, HRE-TKeGFP), blood flow/permeability (Hoechst 33342), micro-vessels (CD31 and SMA), apoptosis (cleaved caspase 3) and hypoxia response (CA9). After bortezomib administration, overall apoptosis index was significantly increased and blood perfusion was dramatically decreased in tumor xenografts. More importantly, apoptosis signals were found preferentially located in moderate and severe pretreatment hypoxic regions in both tumor and endothelial cells. Meanwhile, DCE MRI examinations showed that the tumor blood flow and permeability decreased significantly after bortezomib administration. The present study revealed that bortezomib reduces tumor hypoxia response and blood perfusion, thus, presenting antivascular properties. It will be important to determine the hypoxic/perfusion status pre- and during treatment at further translational studies.

## INTRODUCTION

Targeting the ubiquitin-proteasome pathway has emerged as a rational approach in the treatment of human cancer [1, 2]. Bortezomib (Velcade, Millennium-The Takeda Oncology Co, Boston, MA) is the first proteasome inhibitor approved by the US Food and Drug Administration (FDA) for the treatment of newly-diagnosed or relapsed/refractory multiple myeloma and mantle cell lymphoma [3, 4]. It represented a significant milestone in the treatment of multiple myeloma, which is second most common hematologic cancer in the United States (after Non-Hodgkin Lymphoma); an estimated 24,050 new cases and 11,090 deaths in the USA in 2014 [5].

There are multiple tumor types that exhibit characteristics associated with a dysregulated ubiquitin-proteasome system, including non-small cell lung cancer, colorectal cancer, and others, which could potentially benefit from proteasome inhibitor therapy [6–8]. Phase I-II trails for solid tumors were conducted with either bortezomib alone or combined with radiotherapy or chemotherapy for colorectal cancer, head and neck cancer and non-small cell lung cancer [9–11]. However, expansion of bortezomib in the treatment of the more complex solid tumors has been less successful [8]. Therefore, it is important to clarify the critical biological mechanism, recognize the early response, and select the patient responsive to bortezomib and other proteasome inhibitors for a successful therapy.

Both clinical and preclinical data suggested that therapeutic advantage of bortezomib in patients with solid tumors could be from the strong inhibition of hypoxia-inducible factor-1 (HIF-1) response [9, 12]. Hypoxia response is an important protection mechanism for survival of hypoxic tumor cells [13]. HIF-1, a heterodimeric complex composed of an O_2_-labile α-subunit (HIF-1α), which is degraded by the proteasome under aerobic conditions, and a stable β-subunit (HIF-1β), mediates the primary transcriptional response to hypoxic stress in tumor cells [14]. Although bortezomib blocks the degradation of HIF-1α in the proteasome, the accumulating HIF-1α is unable to activate an effective hypoxia response, rendering tumor cells vulnerable to a hostile hypoxic microenvironment [12]. Importantly, the cytotoxicity of bortezomib was found preferential toward not only tumor cells but also endothelial cells under the hypoxic condition *in vitro* [15, 16]. Since hypoxia is one of the fundamental characteristics of solid tumors [17], it might be feasible to improve the efficacy of bortezomib taking advantage of its inhibition effect on hypoxia response.

In this study, we aimed to 1) investigate the role of pretreatment tumor hypoxic status on the effect of bortezomib treatment and the effects of bortezomib on tumor microcirculation; 2) explore the feasibility of using DCE MRI to noninvasively evaluate the biological effects of bortezomib.

## RESULTS

### Bortezomib effectively inhibits HIF-1 hypoxia response *in vitro*

**Figure 1:**
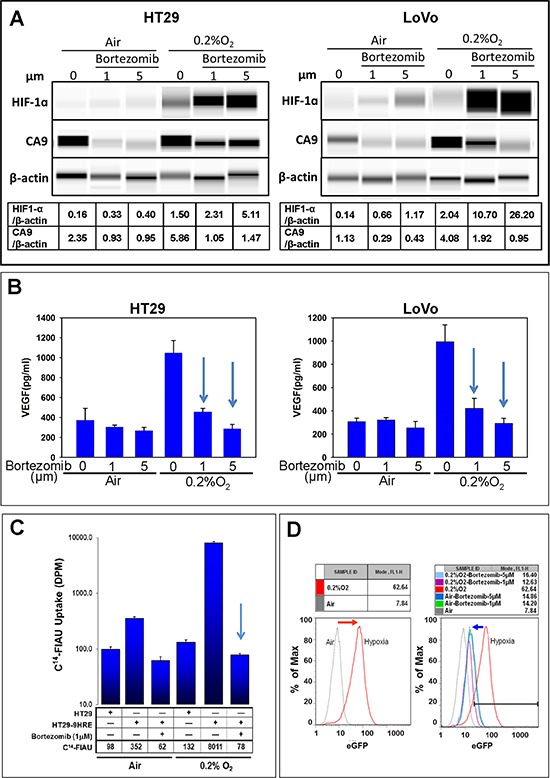
HIF-1α mediated hypoxia response inhibited by bortezomib Cells were exposed to bortezomib for 24 h under 20% O_2_) or 0.2% O_2_, and the following assays were performed. **A.** Western Blot (HIF-1α and CA9). **B.** ELISA (VEGF concentration in tumor cell-conditioned medium). **D.** Flow cytometry analyses (eGFP). **C.** Liquid scintigraphy counter (uptake of ^14^C-FIAU for TK function). The experiments were performed at least thrice and representative results are shown. Arrow: *p* < 0.05

In untreated cells, the expression of HIF-1α and its downstream proteins, CA9 and VEGF (extracellular), in HT29 and LoVo cells were significantly higher under hypoxia (0.2% O_2_) than under normoxia (20% O_2_) (Fig. 1A and 1B). Similarly, the expression of hypoxia response reporter gene eGFP was increased (Fig. 1D), and the function of reporter gene TK was also enhanced under hypoxia (Fig. 1C) in HT29 cells carrying HRE-TK/eGFP.

With bortezomib treatments at indicated doses (1 μM and 5 μM), HIF-1α subunits in HT29 and LoVo cells were protected from proteasomal degradation under both normoxic and hypoxic conditions (Fig. 1A). Cellular protein levels of CA9 were strongly inhibited by bortezomib, in both under normoxia and hypoxia (Fig. 1A). Excreted VEGF levels decreased significantly in response to bortezomib treatment (Fig. 1B). The hypoxia-induced expression of eGFP reporter gene was significantly decreased after bortezomib treatments (Fig. 1D), and the TK function at 0.2% O_2_ condition was also suppressed more than 100 folds (Fig. 1C). These data validated that bortezomib could effectively interfere with the HIF-1 hypoxia response.

### Bortezomib induces apoptosis and decreases tumor blood flow in xenografts

**Figure 2:**
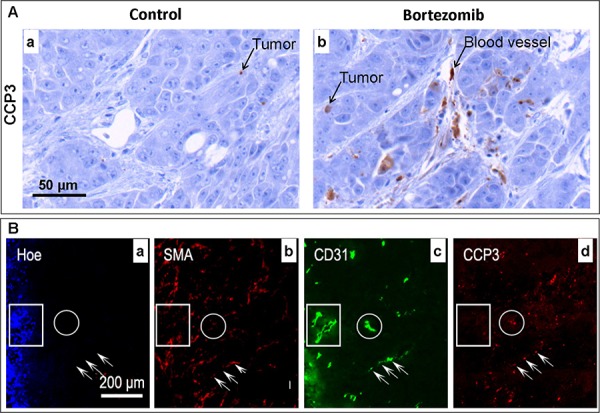
Effects of bortezomib on HT29–9HRE tumors assessed *ex vivo* by immunohistochemistry (IHC) Nude mice bearing HT29–9HRE flank tumors were given bortezomib (2 mg/kg) or 1x PBS. Tumors were dissected and stained using IHC. **A.** Apoptosis marker CCP3 (arrows) IHC images for the PBS (control)- (a) and bortezomib-treated tumors (b). **B.** Multiple markers IHC images. a: blood flow: Hoechst 33342 (Hoe, blue). b: pericyte: SMA (red). c: endothelial cell: CD31 (green). d: CCP3 (red). Rectangular regions: mature microvessels (SMA positive), resistant to bortezomib. Round circles and arrows: immature microvessels, sensitive to bortezomib.

Significant apoptosis was induced in HT29–9HRE xenografts at 24 h after the bortezomib treatment, with a higher CCP3 positive percentage (5.26% ± 0.98%) in the bortezomib group (*n* = 8) than in the control group (0.58% ± 0.13%, *n* = 8), *p* < 0.001. As shown in Fig. 2A-b, intense positive CCP3 signals were found not only in tumor cells (1.98% ± 0.41%) but also in endothelial cells (3.28% ± 0.63%), *p* < 0.01. However, the CCP3 positive signal was barely detectable in the control tumor (Fig. 2A-a).

The overall tumor blood perfusion was dramatically decreased after the bortezomib treatment, as Hoechst 33342 positive percentage decreased from 18.72% ± 2.59% in the control group to 3.57% ± 0.83% in the bortezomib group, *p* < 0.001. The microenvironment of the tumor after bortezomib treatments was demonstrated by a representative section shown in Fig. 2B, with multiple images of IHC staining of blood flow, blood vessel and apoptosis. Within the well perfused area (strong positive Hoechst 33342 signals, the rectangular frame, 2B-a) and the poorly perfused area (no Hoechst 33342 signal, the round circle, 2B-a), the micro-vessels were all lined with endothelial cells (CD31+, 2B-c) surrounded by pericytes (SMA+, 2B-b). However, strong apoptosis signals (CCP3+) were only found in the non-perfused endothelial cells (arrows, 2B-d).

### The effect of bortezomib was dependent on the pretreatment tumor hypoxia status

**Figure 3:**
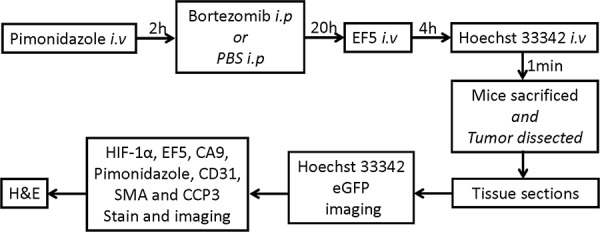
Experiment flowchart for bortezomib administration, multiple marker injection and IHC staining

**Figure 4:**
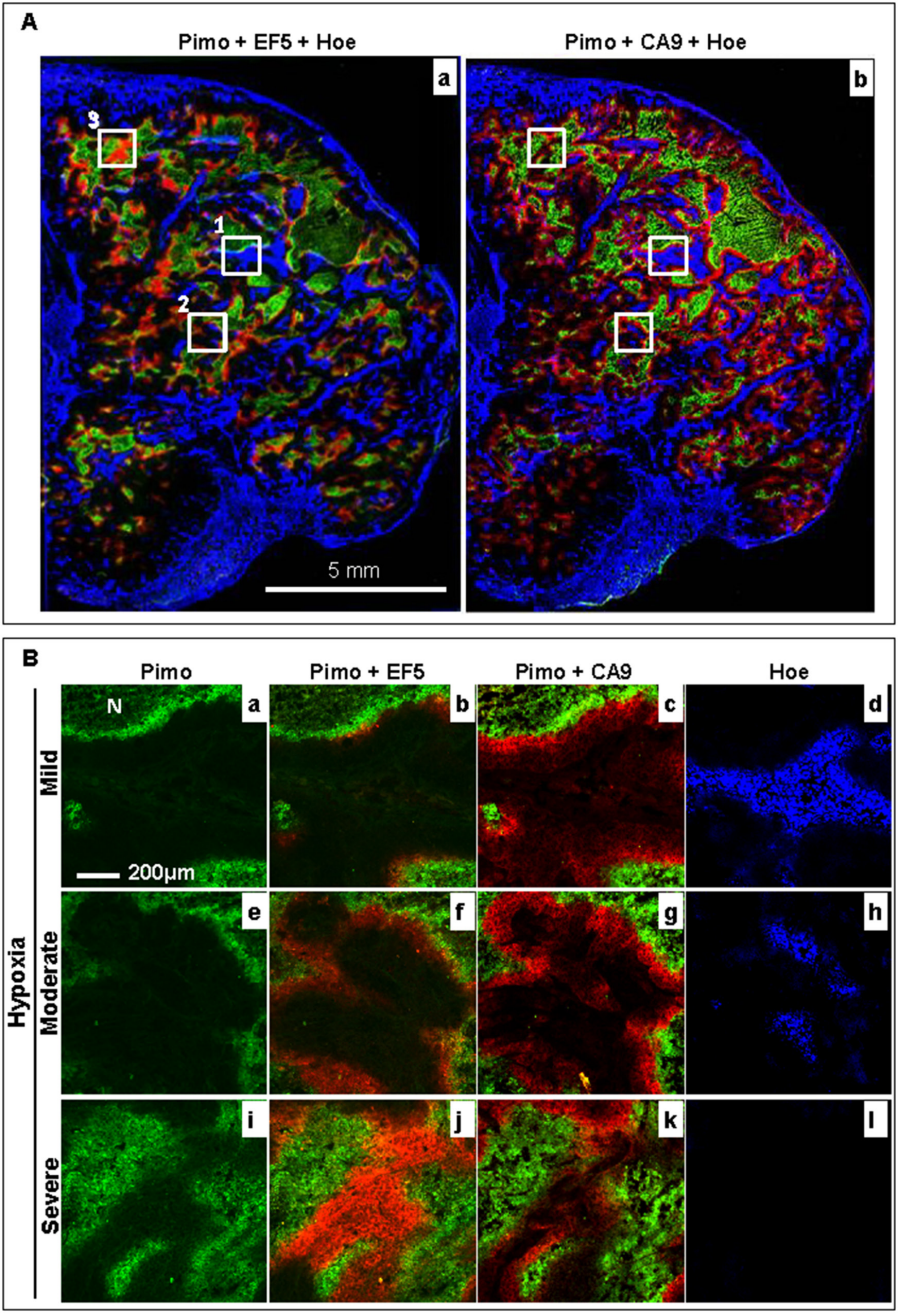
Changes in the hypoxia microenvironment in a representative control HT29–9HRE tumor **A.** Composite image of pimonidazole (green), EF5 (red) and Hoechst 33342 (blue) (a), and pimonidazole (green), CA9 (red) and Hoechst 33342 (blue) (b). Solid squares 1, 2, and 3 represent the mild (<10% pimonidazole positive), moderate (<20% pimonidazole positive), and severe (>20% pimonidazole positive) hypoxic regions, respectively. **B.** Enlarged images of square 1 (a~d), 2 (e~h) and 3 (i~l). Pimo: pimonidazole, Hoe: Hoechst 33342

Changes in the tumor hypoxia status, the hypoxia response of cells, and the blood flow within a 22 h interval was firstly been characterized with the rigid protocol (Fig. 3). The results from a representative control tumor are shown in Fig. 4. It was found that the original hypoxic tumor cells, labeled by the first hypoxia marker pimonidazole (green), were located near the necrotic zone (identified by adjacent H&E staining, data not shown), and the newly developed hypoxic tumor cells, labeled by the second hypoxia marker EF5 (red) given 22 h later, emerged near the perfused blood vessel (blue Hoechst 33342 signal, Fig. 4A-a). Almost all newly developed hypoxic tumor areas labeled by EF5 (red, Fig. 4A-a) showed the apparent expression of hypoxia response protein CA9 (red, Fig. 4A-b).

To investigate the correlation between hypoxia status and response to bortezomib treatment, the entire tumor microscopic images from eight tumors in each group were divided into a batch of 1 × 1 mm^2^ regions in which the hypoxia level were classified into three grades based on the percentage area staining positive for pimonidazole: mild (<10%), moderate (>10%), and severe (>20%), respectively. It was demonstrated as representative areas shown as solid squares 1, 2 and 3 in Fig. 4A, respectively. As magnified images shown in Fig. 4B, while baseline hypoxia extent increased (pimonidazole positive percentages: 8%, 12%, and 38%. a, e and i), the local tumor blood flow gradually decreased (Hoechst 33342 positive percentages: 25%, 8%, and 0%. d, h and l). Meanwhile, the ratio of newly developed hypoxic cells gradually increased (only EF5 positive percentages: 3%, 11%, and 32%. b, f and j). However, the hypoxia response did not increase correspondingly (CA9 positive percentages:11%, 13%, and 8%. c, g and k).

**Figure 5:**
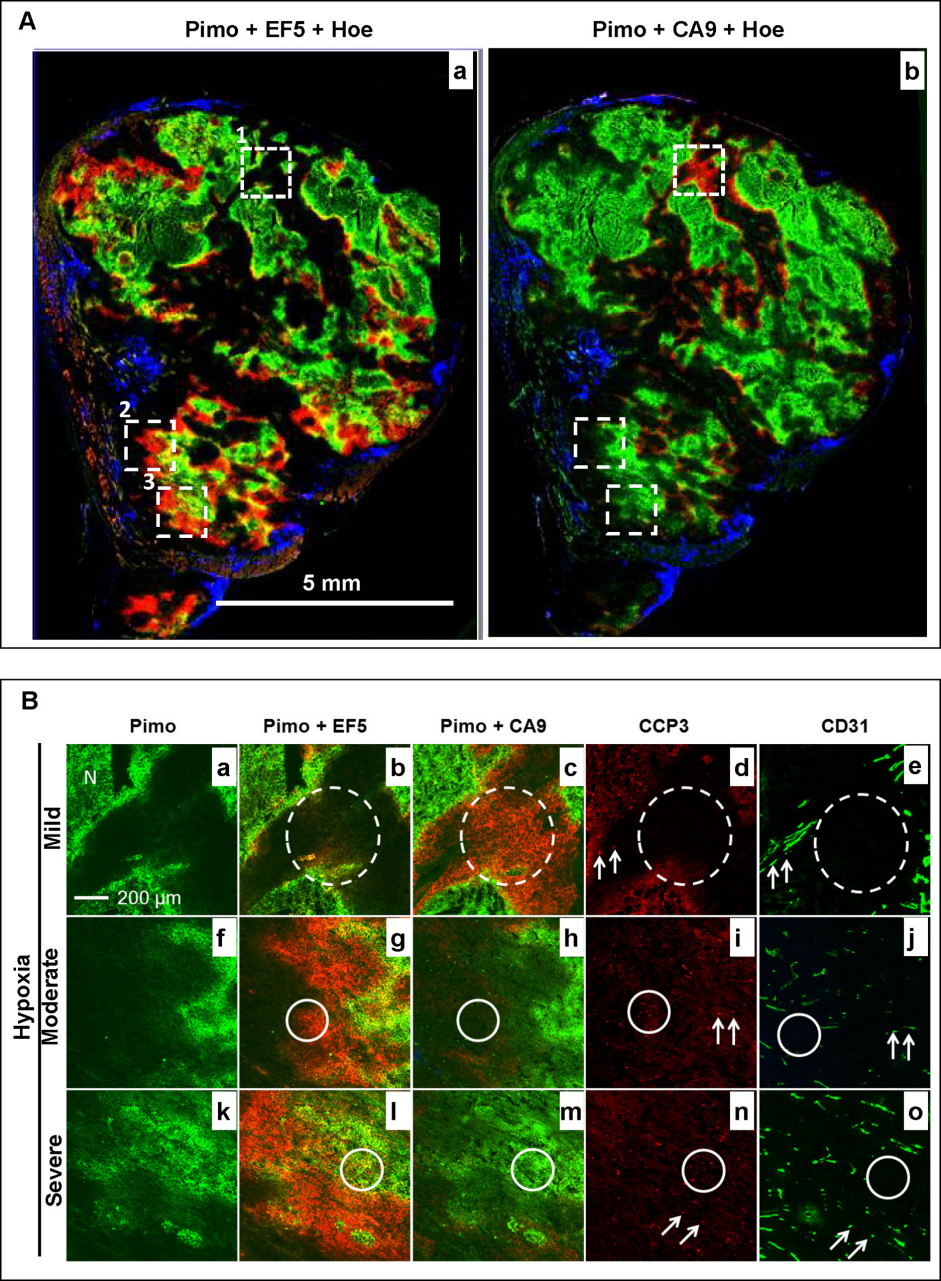
Changes in the hypoxia microenvironment in a representative bortezomib-treated HT29–9HRE tumor **A.** Composite image of pimonidazole (green), EF5 (red) and Hoechst 33342 (blue) (a), and pimonidazole (green), CA9 (red) and Hoechst 33342 (blue) (b). Dashed squares 1, 2, and 3 represent the mild (<10% pimonidazole positive), moderate (<20% pimonidazole positive), and severe (>20% pimonidazole positive) hypoxic regions, respectively. **B.** Enlarged images of square 1 (a~d), 2 (e~h) and 3 (i~l). Dotted-line circles: region without apoptosis (bortezomib resistant). Solid-line circles and arrows: region with apparent apoptosis (bortezomib sensitive). Pimo: pimonidazole, Hoe: Hoechst 33342, CCP3: cleaved caspase 3, N: Necrosis

In contrast, after bortezomib treatments, the relationships between tumor hypoxia and hypoxia response does not exist any more (Fig. 5). There was almost no apparent blood perfusion in the central regions, with only few Hoechst 33342 positive signals in the peripheral regions (Fig. 5A). The total tumor blood perfusion significantly decreased in treated tumors, with Hoechst 33342 positive percentage of 3.57% ± 0.83% in comparison to 18.72% ± 2.59% in the control group, *p* < 0.001. Two major patterns of the relationship between hypoxia status and hypoxia response were found. At region with mild pretreatment hypoxia (pimonidazole+, green, dashed square 1 in Fig. 5A and Fig. 5B-a~e), there was no *de novo* hypoxic populations (EF5+, red) but apparent CA9 expression (red). However, at regions with moderate (dashed square 2 in Fig. 5A and 5B-f~j) and severe pretreatment hypoxia (dashed square 3 in Fig. 5A and 5B-k~o), there were *de novo* hypoxic cells (EF5+) around original hypoxic cells (pimonidazole+), but no CA9 expression.

In addition, it was also found that the differences in the microvasculature structures and the heterogeneous distribution of pretreatment hypoxia led to the significant diverse effect of bortezomib (Fig. 5B). In the mild hypoxia regions, the majority of the micro-vessels (CD31+) were relative large and formed with discernible lumen (Fig. 5B-e). In the contrary, the microvasculature was mainly in dot or short line shape without discernible lumen formation in the moderate (Fig. 5B-j) and severe hypoxic regions (Fig. 5B-o). The apoptosis signals were found preferentially located in moderate and severe pretreatment hypoxic regions. The CCP-3 positive percentage in these regions was 7.54% ± 1.31% and 7.92% ± 1.12%, respectively, higher than that in mild hypoxic regions (1.08% ± 0.32%), *p* < 0.001. The apoptosis was found both in tumor cells (2.57% ± 0.64% and 2.63% ± 0.59%, solid circles) and endothelial cells (4.97% ± 1.53% and 5.29% ± 1.48%, arrows) in moderate and severe hypoxic regions, respectively. However, there were only CCP3 positive signal in endothelial cells (1.08% ± 0.32%, arrows), but not in tumor cells (dashed circle) in the mild hypoxia region.

### DCE MRI can effectively monitor effects of bortezomib *in vivo*

**Figure 6:**
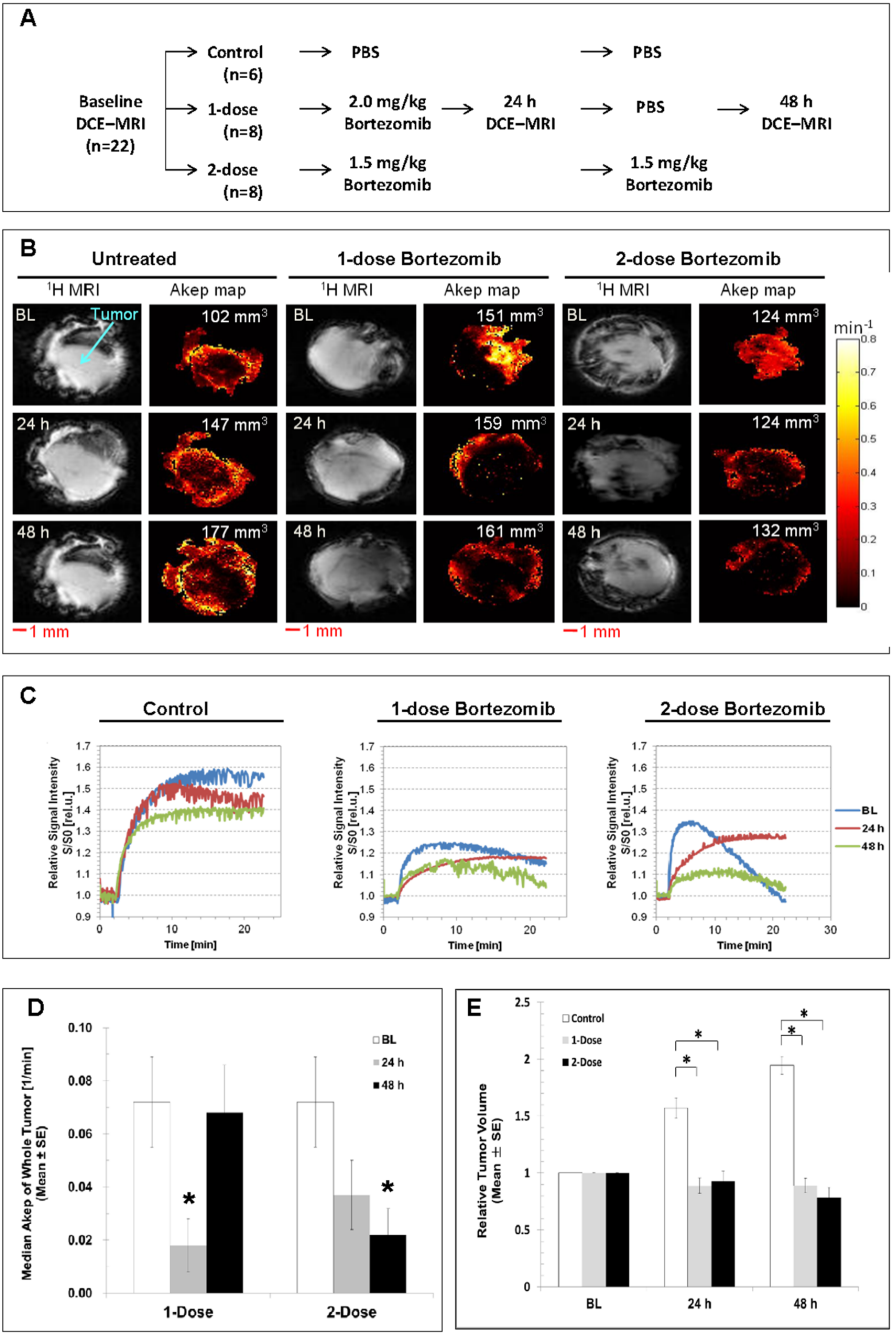
*In vivo* DCE MRI of subcutaneous HT29–9HRE xenografts **A.** Experiment flowchart. **B.** Representative ^1^H MR single-slice images and corresponding Ak_ep_ pseudocolor images at baseline, 24 h and 48 h for the control, 1-dose and 2-dose groups. **C.** Representative buildup time-signal intensity curves of a single representative tumor voxel at baseline, 24 h and 48 h for the control, 1-dose and 2-dose groups. **D.** Whole-tumor median Ak_ep_ values. The baseline values were averaged for all tumors and plotted beside those at 24 h and 48 h after 1-dose or 2-dose of bortezomib administration. **E.** Tumor volumes normalized to baseline at 24 h and 48 h for untreated and treated tumors. *p < 0.05

Serial DCE MRI scans were performed with the protocol depicted in Fig. 6A. Representative ^1^H weighted MR images and corresponding Ak_ep_ maps in pseudo color were shown for each treatment arm in Fig. 6B. The Ak_ep_ maps show that vascular perfusion can be evaluated and is spatially heterogeneous at the baseline DCE MRI. There was no discernible change in vascular flow/perfusion in the control tumor group over the time interval (*n* = 6). In the single-dose group (*n* = 8), the tumor vascularity was dramatically reduced in both center and peripheral tumor regions at 24 h, and slightly recovered from the periphery to the center at 48 h. However, in the two-dose group (*n* = 8), the tumor blood flow and permeability was continuously decreased in both peripheral and central regions at 24 h and 48 h after the bortezomib administration. Representative buildup time-signal intensity curves from single tumor voxels at the baseline, 24 h, and 48 h after treatments for each group are displayed in Fig. 6C. In the control group (*n* = 6), there were no significant changes in the initial slopes (Ak_ep_) of the curves at 24 h and 48 h compared to the baseline curve, suggesting no significant change in local blood flow and permeability. However, in the single- and two-dose groups, Ak_ep_ of the 24 h and 48 h curves were lower than those at baseline, suggesting that the local blood flow and permeability decreased post-treatment.

The dynamic changes in average tumor blood flow and permeability (whole-tumor median Ak_ep_ values) were shown in Fig. 6D. To eliminate the bias from inter-tumoral heterogeneous baseline vascular blood flow and permeability, baseline whole-tumor Ak_ep_ values were pooled from all three groups to evaluate vascular changes in response to bortezomib treatment. It was found that the average whole-tumor median Ak_ep_ values were significantly reduced from 0.072 ± 0.018/min at baseline (*n* = 22) to 0.018 ± 0.010/min at 24 h and recovered at 48 h (0.068 ± 0.017/min) after single-dose bortezomib treatments (gray bars, *n* = 8), and continuously decreased at 24 h (0.037 ± 0.014/min) and 48 h (0.022 ± 0.010/min) after two consecutive treatments (black bars, *n* = 8), *p* < 0.05. During the 3-day time frame, the control tumors grew rapidly. In contrast, a significant growth delay was observed in both, the one-dose and two-dose groups (Fig. 6E).

### DCE MRI could indirectly reflect the biological effect of bortezomib

**Figure 7:**
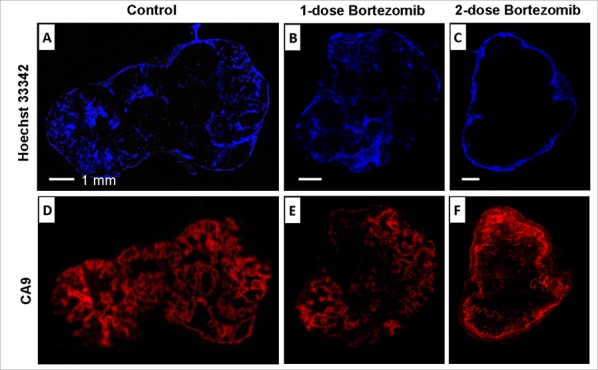
*Ex vivo* microscopic images After the last DCE MRI, nude mice were *i.v*. injected with Hoechst 33342 and sacrificed 1 min later. Hoechst 33342 (A, B, C) and CA9 (D, E, F) were shown for representative tumors from control, 1-dose and 2-dose groups.

*Ex vivo* data validated that the effects of bortezomib on tumor vascularity paralleled its effect on the tumor hypoxia response (Fig. 7). The effect of bortezomib on tumor perfusion (Hoechst 33342) was similar to the effects on *in vivo* tumor blood flow and permeability as assessed by DCE MRI. Similar results were obtained when evaluating the extent of hypoxic areas in the tumor at two time points *ex vivo* by immunohistochemistry of pimonidazole and imaging of eGFP (data not shown). The distribution of Hoechst 33342 at 50 h after single-dose bortezomib treatments was observed in both central and peripheral tumor regions (Fig. 7B), which suggested that the effect of bortezomib on tumor perfusion has vanished or decreased. In the two-dose group, tumor perfusion was significantly decreased with only Hoechst 33342 detected in the peripheral regions (Fig. 7C). In addition, the effect of bortezomib on tumor hypoxia response (CA9) was similar as its effects on tumor blood flow and permeability. The expression of CA9 was recovered approximately to the level of the control group at 50 h after single-dose bortezomib (Fig. 7E), but dramatically suppressed with two-dose treatments (Fig. 7F).

## DISCUSSIONS

As bortezomib is still in early phase of clinical trail for treating solid tumors, it is key to clarify its critical biological effects and mechanism and select the patients responsive to the treatment. In the present study, we found that the pretreatment tumor hypoxia microenvironment plays a critical role in the effects of bortezomib. Higher apoptotic indexes were found in the endothelial cells and tumor cells in severe hypoxic regions, which were closely related to the inhibition/reduction of tumor hypoxia response. Noninvasive DCE MRI could effectively monitor the changes in tumor vascularity, linked to tumor hypoxia [18], reflecting the biological effects of bortezomib. Our data thus support, that the an early response to bortezomib treatment may have the potential to select patients who would most benefit from bortezomib treatment and establish a rational scheme for successful targeted therapy.

The hypoxia pathway targeting effect of bortezomib has been studied previously in different models [12, 15, 19–21]. *In vitro* and *in vivo* data showed that bortezomib protected HIF-1α from proteasome degradation and impaired its function in cervical cancer [12]. Biphasic effects of proteasome inhibitors on the stability of HIF-1α have been also found [20]. Fels *et al.* found that bortezomib exhibited significantly higher cytotoxicity toward hypoxic than normoxic tumor cells, which was accompanied by enhanced activation of an unfolded protein response [15]. In present study, it was found that the effect of bortezomib was dependent on the status of pretreatment tumor hypoxia, as assessed by the analysis of multiparametric *ex vivo* images (Fig. 4). The cytotoxicity of bortezomib toward tumor cells only occurred in areas that were, at pretreatment, moderately to severely hypoxic. It could be closely related to bortezomib's inhibitory effect on the tumor hypoxia response. Apoptosis was only activated in hypoxic tumor areas without associated expression of CA9, a protein expressed downstream of the primary hypoxia response characterized by HIF-1 stabilization. In addition, it was also found that spatial microvascular heterogeneity and the heterogeneous distribution of pretreatment hypoxia were associated with a significant and equal spatially diverse inhibitory effect of bortezomib on the hypoxia response (Fig. 5). Therefore, it is worth to further explore the dual antitumor mechanism of bortezomib in solid tumor, although clinical data showed that solid tumors of various histological origins were resistant to either bortezomib as single treatment or in combination with radiotherapy or chemotherapy [6–9]. Our data suggest that preexisting hypoxia plays an important role in antitumor efficacy of bortezomib, and that the inhibition or reduction of the HIF-1 hypoxia response pathway promotes apoptosis in hypoxic tumor cells.

More importantly, our study demonstrated that pretreatment hypoxia also plays a critical role in the effects of bortezomib on the tumor vasculature. Tumor endothelial cells within poorly developed microvasculature were more sensitive to bortezomib than tumor cells, accounting for ~62.35% of the apoptotic population (Figs. 2 and 5). Previous *in vitro* experiments found that endothelial cells exposed to bortezomib undergo death to an extent that depends strictly on their activation state [22]. Endothelial cells under hypoxic condition or actively proliferating are sensitive to bortezomib, which activates autophagy or apoptosis [16, 22]. Preexposing endothelial cells to hypoxia to induce the expression of HIF-1α protein, will greatly enhance the proapoptotic effect of bortezomib [16, 22, 23]. Bone marrow angiogenesis is emerging as a critical component of multiple myeloma development and progression, and hence, provides an attractive therapeutic target for the disease [24]. Bortezomib has shown antiangiogenic properties in multiple myeloma via direct and indirect effects on endothelia cells [25]. When HUVECs were induced to express HIF-1α prior to bortezomib treatment *in vitro*, proliferative and angiogenic responses were abolished, and a greatly enhanced proapoptotic effect was promoted [23]. These results indicate that HIF-1α up-regulation may sensitize endothelial cells to the antiangiogenic and proapoptotic effects of bortezomib and explain the histological findings in the current study.

As bortezomib induced apoptosis in tumor endothelial cells, it led to the collapse of corresponding vessel functionality, and significantly decreased localized and overall tumor vascularity. This was demonstrated by both *ex vivo* imaging of tumor sections (Figs. 5 and 7) and *in vivo* DCE MRI (Fig. 6). It was found that the inhibition of tumor microcirculation by bortezomib was synchronized with its inhibitory effect on hypoxia response (as assessed by decreased expression of eGFP and CA9). When tumor vascularity was decreased by bortezomib, the hypoxia response was also reduced, and vice versa. Therefore, using DCE MRI to monitor the effect of bortezomib on tumor vascularity could effectively reflect its biological effects on the histological level. It provides thus a potent clinical tool to early and noninvasively select patients responsive to bortezomib treatment and establish a rational scheme for combining bortezomib with other therapeutic approaches. Preclinically, DCE MRI has been used extensively as a noninvasive tool to assess tumor blood perfusion and predict treatment response [26, 27]. Using DCE MRI and high-resolution computed tomographic imaging of vascular casts, the vasculature of xenograft tumors has been characterized and prominent differences in vessel perfusion, permeability, and architechture have been identified and ultimately resulted in optimizing bortezomib exposure to improve treatment efficacy [28]. Clinically, DCE MRI has been used in early clinical development of vascular directed anticancer therapies over the last decade and has been proven helpful in assessing whether mechanistic goals are achieved, in assisting dose selection, in selecting subpopulations enriched for response and in predicting patient benefit [29].

In summary, the present study revealed that bortezomib significantly inhibited the hypoxia response pathway in tumors and reduced tumor vascular blood flow and permeability. Our results emphasize the importance of determining the spatial extent and heterogeneity of hypoxia and tumor vascularity pre- and during treatment for translational studies. Although limited by small sample size, (12 of 91 patients were observed), DCE MRI has already been employed in a recently reported phase I study, treating advanced, refractory malignancies with a combination of bortezomib plus anti-angiogenic therapy (bevacizumab) [30]. Additionally, a phase I/II study to evaluate efficacy and safety of using bortezomib in combination with chemotherapy and bevacizumab as the first-line treatment in advanced non-small cell lung cancer has also been conducted recently in patients [31]. Our results, together with other clinical and preclinical studies together, demonstrate the significant advantages of conducting translational studies including treatments targeting the proteasome in human solid tumors accompanied by *ex vivo* and *in vivo* imaging.

## MATERIALS AND METHODS

### Reagents and antibodies

Bortezomib was purchased from Millenium Pharmaceuticals (Cambridge, MA). Pimonidazole HCl (Hypoxyprobe-1) and its antibody were purchased from Natural Pharmacia International, Inc. (Burlington, MA). 2-(2-nitro-1H-imidazole-1- yl)-N-(2,2,3,3,3-penta-fluoropropyl) acetamide (EF5) and its antibody were kindly provided by Dr. Cameron J. Koch (University of Pennsylvania, Philadelphia, PA). Hoechst 33342 trihydrochloride was purchased from Sigma-Aldrich (St Louis, MI). [^14^C] 2′ -Deoxy-2′ -fluoro-β-D-arabinofuranosyl-5-iodouracil (^14^C-FIAU, 0.025μCi/mL, chemical purity of 97.7%) was purchased from Moravek Biochemicals (Lane Brea, CA). The antibodies used were against HIF-1α (Chemicon, Billerica, MA), cleaved caspase 3 (CCP3, Cell Signaling Technology, Danvers, MA), CD31 (BD Biosciences, San Jose, CA) and α-smooth muscle actin (α-SMA, Sigma-Aldrich). The antibody against carbonic anhydrase 9 (CA9, cG250) was kindly provided by Dr. Gerd Ritter (Ludwig Institute for Cancer Research, New York, NY). The secondary antibodies for immunofluorescence staining were matched Alexa Fluor fluorescent dye-conjugated antibodies (Cell Signaling Technology). VEGF ELISA kit was purchased from TSZ ELISA (Waltham, MA).

### Cell culture

Human colorectal carcinoma HT29 and LoVo cells were maintained in McCoy's 5A medium or Dulbecco's Modified Essential Medium (DMEM), respectively, supplemented with 10% fetal bovine serum and antibiotics at 37°C and 5% CO_2_ in humidified air. Previously developed HT29 cells, carrying a 9 hypoxia-response elements (9HRE) driven HSV1-TK/eGFP (designated as HT29–9HRE), were maintained as described previously [32]. Stable hypoxia-inducible gene expression in HT29–9HRE cells was periodically tested by PCR and flow cytometry. Cells for experiments were obtained from recently thawed vials and used below passage 10.

### *In vitro* response to bortezomib

HT29–9HRE cells were treated at 37°C with bortezomib at indicated concentrations and exposed to 0.2% O_2_, 5% CO_2_, 94.8% N_2_ (hypoxia) in an *In vivo* 2-400 Hypoxia Workstation (Biotrace, Bridgend, UK) or maintained in humidified air containing 5% CO_2_ for 24 h. Cell lysates were prepared for western blot (HIF-1α and CA9), which was performed using a Sally Simple Western instrument (ProteinSimple) [33]. VEGF concentrations in tumor cell-conditioned media were determined by ELISA (Bio-rad). The expression of eGFP in live HT29–9HRE cells was examined by flow cytometry, and TK function by measuring ^14^C-FIAU uptake, as described by us previously [32].

### Animal experimental procedure

All animal protocols were approved by the Institutional Animal Care and Use Committee. HT29–9HRE xenografts were formed by injecting 5 × 10^6^ cells *s.c*. into the hind limbs or the right flank (for DCE MRI) of 6–8 weeks old female nude mice (NCr athymic nu/nu, National Cancer Institute Frederick Cancer Research Institute). Unless indicated otherwise, nude mice, bearing HT29–9HRE xenografts, were injected *i.v*. with the first hypoxia marker pimonidazole (80 mg/kg), 2 h later injected *i.p*. with bortezomib (2 mg/kg) or PBS, 20 h later injected *i.v*. with the second hypoxia marker EF5 (24 mg/kg), and 4 h later injected *i.v*. with perfusion marker Hoechst 33342 (25 mg/kg), followed 1 min later by animal sacrifice. Immediately after sacrifice, tumor tissue was excised, frozen, and embedded in optimal-cutting-temperature medium (OCT). Contiguous frozen tissue sections were cut at a thickness of 8 μm and stored at −80°C. The experimental flowchart was shown in Fig. 3.

### Immunohistochemical staining, fluorescence microscopy, and image analysis

Whole tumor sections were scanned using an Axiovert 200M Inverted fluorescence microscope (Carl Zeiss, Oberkochen, Germany), equipped with a computer-controlled motorized stage, a digital camera, and Metamorph software. All images were acquired at 50× magnification, unless indicated otherwise. Composite images were generated from individual microscope images using the software. Sections were first imaged for eGFP and Hoechst 33342. Then the same or adjacent sections were stained for HIF-1α, pimonidazole, EF5, CA9, CD31, SMA and CCP3 according to the manufacturer's instructions, and imaged at the appropriate wavelength for green and red fluorescence, respectively. Lastly, the sections were stained with H&E according to standard protocol.

The analysis included the entire viable tumor area identified on the H&E slide. The images were carefully adjusted and analyzed by Adobe Photoshop 7.0. For each image, the threshold was set individually at a brightness level that best separated the signal from the background. Based on the threshold images, the percentages of positive stained area were measured for each marker.

### Dynamic contrast enhanced ^1^H MRI (DCE MRI)

DCE MRI was performed on a Bruker 7T BioSpin MR spectrometer (Bruker, Germany) with a home-built, solenoid ^1^H MR coil. Sagittal slices of interest were identified from a pilot MR scan. The mouse tail vein was catheterized to facilitate administration of the MR contrast agent Gd-DTPA (0.1 mM Gd/kg, Magnevist^®^, Berlex Laboratories, Wayne, NJ). The mice were anesthetized with isoflurane (0.8%–1.5%) in oxygen (1.5 ml/min) with respiration monitored. T_1_-weighted DCE MRI (Fast Low Angle Shot (FLASH) MRI, 4 slices, 1 mm slice thickness, 1 average (number of averages, NA), 312 repetitions (number of repetitions, NR), 3.2 ms echo time (TE), 34 ms repetition interval (TR), 128 × 128 matrix, 15 mm × 15 mm field of view (FOV), ~45° flip angle, 22 min acquisition time) was performed at 4.3 s temporal resolution and 117 μm × 117 μm in-plane resolution.

After the baseline DCE MRI, the animals were randomly assigned into three groups: 1) control (PBS), 2) 1-dose bortezomib (2.0 mg/kg), or 3) 2-dose bortezomib (1.5 mg/kg administered 24 h apart). Each animal underwent three sequential DCE MRI experiments (day 0, 1, and 2) with spin density MR images facilitating the tumor slice alignment of the baseline DCE MRI and with the subsequent DCE MRI performed at 24 h and 48 h later. The time-signal curves, obtained by DCE MRI, were normalized with respect to the data without contrast agent and fitted voxel-by-voxel using the Hoffman model [18, 34]. Ak_ep_ maps were generated for the corresponding tumor slices for all 3 time points. To quantify the perfusion (tumor blood flow and permeability) changes due to bortezomib treatments, the median Ak_ep_ value of each tumor was calculated from whole-tumor Ak_ep_ histograms.

Each tumor was measured daily with a digital caliper in three orthogonal dimensions (a, b and c). Tumor volume was calculated as πabc/6 and compared between groups. For *ex vivo* evaluation of hypoxia and perfusion changes in response to treatment, mice were injected *i.v. with* pimonidazole after the last DCE MRI, 2 h later injected *i.v*. with Hoechst 33342, and 1 min later sacrificed. Histological analysis was performed as described above and compared with the DCE MRI results.

### Statistical analysis

Statistical analysis was performed using SPSS 16.0. Data were shown as mean ± SE. Differences in the markers *ex vivo* among treatment groups were assessed using 2-tailed Student's *t* test, ANOVA or the Mann-Whitney rank-sum test. A *P* value less than 0.05 was considered statistically significant.
